# Crystallographic characterization of two novel crystal forms of human insulin induced by chaotropic agents and a shift in pH

**DOI:** 10.1186/1472-6807-7-83

**Published:** 2007-12-19

**Authors:** Mathias Norrman, Gerd Schluckebier

**Affiliations:** 1 Diabetes Protein Engineering, Novo Nordisk A/S, Novo Nordisk Park, DK-2760 Måløv, Denmark; 2 Molecular Biophysics, Lund University, Box 124, SE-22100 Lund, Sweden

## Abstract

**Background:**

Insulin is a therapeutic protein that is widely used for the treatment of diabetes. Its biological function was discovered more than 80 years ago and it has since then been characterized extensively. Crystallization of the insulin molecule has always been a key activity since the protein is often administered by subcutaneous injections of crystalline insulin formulations. Over the years, insulin has been crystallized and characterized in a number of crystal systems.

**Results:**

Interestingly, we have now discovered two new crystal forms of human insulin. The crystals were obtained when the two chaotropic agents, urea and thiocyanate were present in the crystallization experiments, and their structures were determined by X-ray crystallography. The crystals belong to the orthorhombic and monoclinic crystal systems, with space groups C222_1 _and C2 respectively. The orthorhombic crystals were obtained at pH 6.5 and contained three insulin hexamers in R_6 _conformation in the asymmetric unit whilst the monoclinic C2 crystals were obtained at pH 7.0 and contained one R_6 _hexamer in the asymmetric unit. Common for the two new crystals is a hexamer-hexamer interaction that has not been found in any of the previous crystal forms of insulin. The contacts involve a tight glutamate-glutamate interaction with a distance of 2.3 Å between groups. The short distance suggests a low barrier hydrogen bond. In addition, two tyrosine-tyrosine interactions occupying a known phenol binding pocket contribute to the stabilization of the contacts. Within the crystals, distinct binding sites for urea were found, adding further to the discussion on the role of urea in protein denaturation.

**Conclusion:**

The change in space group from C222_1 _to C2 was primarily caused by an increase in pH. The fewer number of hexamer-hexamer interactions comprising the short hydrogen bond in the C2 space group suggest that pH is the driving force. In addition, the distance between the two glutamates increases from 2.32 Å in the C222_1 _crystals to 2.4 Å in the C2 crystals. However, in both cases the low barrier hydrogen bond and the tyrosine-tyrosine interaction should contribute to the stability of the crystals which is crucial when used in pharmaceutical formulations.

## Background

The therapeutic hormone insulin is a small protein used daily in the medical treatment of diabetes by millions of people. The primary administration route is by subcutaneous injections of microcrystals or mixtures of microcrystals and amorphous protein. After subcutaneous injection, the insulin crystals dissolve slowly, thus leading to a slow intermediate release of insulin into the blood stream. Ever since the biological function of insulin was discovered in the 1920s [1], the molecule has been widely characterized both biophysically and structurally. The crystallographic structure was one of the first protein structures determined [2]. It has since then been crystallized in a number of space groups of which the most common belong to the monoclinic, rhombohedral, cubic and tetragonal crystal forms. The type, size and morphology of the crystals affect how fast insulin is released, which is why crystallization of insulin has been studied extensively. Alternative administration routes are presently a rapidly expanding research field and insulin microcrystals may be well suited for other delivery methods, including pulmonary delivery or sustained release formulations [3-5]. The insulin molecule consists of two chains, A and B, with 21 and 30 residues respectively. Chain A is built up by two helical fragments separated by a short loop linked to one of the helices by an intra-chain disulfide bond. Two additional disulfide bonds link chain A to the larger chain B. In the biologically active form, insulin exists as a monomer in which chain B contains a central helical region flanked by two elongated parts. In the presence of divalent ions like zinc, the monomers assemble into hexamers [6], where each of the two central zinc ions is coordinated by three histidine residues. In the hexameric form, chain B has been shown to exist in two allosteric states denoted T and R [7]. The R state has two allosteric binding sites usually referred to as the phenolic binding site and HisB10 anion site. The T → R state transition and the two different B chain conformations, referred to as T_6 _and R_6_, have been described by spectroscopic and crystallographic studies. The T_6 _conformation, which is characterized by an extended conformation of residues 1–8 of chain B, is obtained at low chloride concentrations and in absence of phenol derivatives [8,9]. Phenolic derivatives are used as preservative in insulin pharmaceutical formulations. The most commonly used are phenol, meta-cresol, resorcinol and methylparaben. The R conformation is obtained in presence of these derivatives and at high chloride concentrations. In this form the first eight residues of the B chain adopt a helical conformation, which together with the central helical segment gives a continuous helix which includes residues B1 to B19 [10-13]. This transition from an extended to an alpha-helical conformation causes the first residue of chain B, PheB1 to undergo a ~30 Å shift in position. Although chloride is the most commonly used anion, other anions such as SCN^-^, OCN^-^, CN^-^, N_3_^- ^and NO_2_^- ^have also been shown to be useful [7,14,15]. Like chloride, in the absence of phenolic derivatives and at high concentrations, these anions are able to induce the R state in three of six monomers in a hexamer. The remaining three monomers have an extended conformation (T state) in the region including residues B1 to B8. The R state of the first three monomers is incomplete with residues B4 to B8 in a helical conformation, while residues B1 to B3 have an extended conformation. This hexamer configuration is denoted T_3_R_3_^f^, where the 'f' indicates a frayed R conformation [16,17].

We here present a study which shows that certain chaotropic additives can induce two novel types of insulin crystals, and that the type of crystals formed depends on the charge state of insulin, i.e. is pH dependent. The structures and crystal packing interaction of the two new crystal forms have been analyzed and compared to crystal packing interactions in other previously known insulin crystals.

## Results and discussion

Crystallization of insulin is of high importance in pharmaceutical formulations and in insulin manufacturing and has been systematically investigated since the 1920s [6,18-20]. By introducing chaotropic agents in the crystallization experiments, we succeeded in identifying two new crystal forms of native human insulin. The crystals were found using two different crystallization experiments. The first crystals were obtained in a crystallization screen with varying concentrations of urea and sodium chloride in presence of zinc and resorcinol. The crystals were initially characterized by X-ray powder diffraction and were shown to have a powder pattern differing from previously known insulin crystal forms [21]. Further optimizations of crystallization conditions resulted in crystals suitable for single crystal analysis and determination of crystal system which was found to be orthorhombic in space group C222_1_. The crystals appeared in the pH range 6.0 – 6.5 while a second type of crystals, characterized as monoclinic with space group C2 grew in the pH range 6.5 – 7.0. In the overlapping pH interval around pH 6.5, the C222_1 _crystals were present in wells with lower salt and urea concentrations. A few drops contained a mixture of the two crystal types. In a parallel experiment, the urea and sodium chloride were substituted for thiocyanate. Interestingly, the same two crystal types appeared here, at the same pH intervals, with a clearer pH distinction at pH 7.0. Crystallization with thiocyanate or chloride ions without a phenol derivative has previously been shown to stabilize the T_3_R_3_^f ^form of hexameric insulin in a rhombohedral crystal system [16,17]. In our case when resorcinol and thiocyanate were present, the orthorhombic C222_1 _and monoclinic C2 crystals appeared. The well characterized monoclinic crystals in space group P2_1 _[10,11] were present at pH values above 7.0 in wells with low salt and high urea concentration and increased in frequency as the pH was raised to become the dominating crystal form at pH ≥ 7.5. The crystals obtained in presence of urea will be referred to as C222_1_urea and C2urea while the two crystal forms obtained with thiocyanate are referred to as C222_1_scn and C2scn.

### Structure of insulin in the orthorhombic lattice

**Figure 1:**
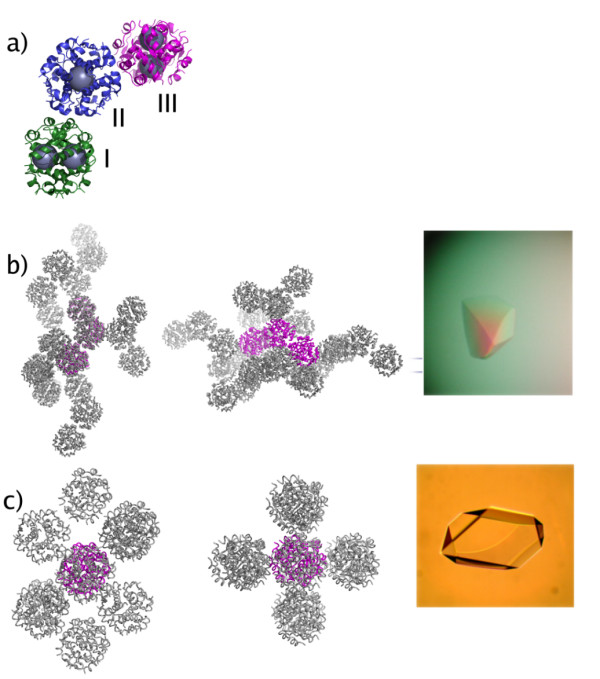
**Crystal packing**. (a) The asymmetric unit of the C222_1 _crystal form viewed down the non-crystallographic three-fold axis of the central hexamer. The flanking hexamers are located around the central hexamer at an angle of ~110°. The local non-crystallographic three-fold axis of the two outer hexamers is almost orthogonal to the central non-crystallographic three-fold axis. The zinc atoms are illustrated as large spheres to mark the position of the three-fold axes. The hexamers are numbered from I to III. (b) The crystal packing in the C222_1 _space group drawn with main chain trace with the asymmetric unit in magenta. (c) The crystal packing of the human insulin in space group C2. The asymmetric unit molecule is colored magenta. The inserts in (b) and (c) show crystals of the C222_1 _and C2 forms, respectively.

The crystals grown at pH 6.5, from both the urea- and thiocyanate (NaSCN) screen, were found to belong to space group C222_1 _with unit cell parameters a = 59 Å, b = 220 Å, c = 223 Å. The asymmetric unit contains three insulin hexamers with a crystal solvent content of 64%. The hexamers have R_6 _conformation and contain two zinc atoms/hexamer coordinated to three histidine residues (HisB10). In the C222_1_urea crystal, the zinc is additionally coordinated by a chloride ion at an average distance of 2.15 ± 0.10 Å, whilst in the C222_1_scn crystal structure the chloride ion is replaced by a thiocyanate. The thiocyanate coordinates to zinc through its nitrogen atom with an average distance of 1.82 ± 0.04 Å. The three hexamers in the asymmetric unit are arranged in an angular formation where the central hexamer connects the two outer hexamers with an angle of ~110°, Figure 1a. The non-crystallographic three-fold axes which pass through the two zinc atoms in each hexamer are almost orthogonal to each other. In both the C222_1_urea and C222_1_scn structures, one co-crystallized resorcinol molecule is bound to each insulin monomer in the phenolic binding pocket. The resorcinol molecule is hydrogen bonded with its first hydroxy group to the carbonyl O atom of CysA6 (average distance 2.6 Å), and the N atom of CysA11 (average distance 2.9 Å). The second hydroxy group hydrogen bonds to a water molecule with an average distance of 2.7 Å. This water molecule forms another hydrogen bond to the O atom of CysA11 with an average distance of 2.8 Å. In the final stages of refinement, one glycerol molecule was modeled into the C222_1_scn structure at a position where it interacted through its oxygen atoms with the amide nitrogen of PheB1 (2.9 Å) and the carbonyl oxygen of ThrA8 (2.9 Å). The crystal packing of the C222_1_urea structure is shown in Figure 1b.

### Structure of insulin in the monoclinic lattice

The crystals obtained at slightly higher pH (pH 7.0) belong to the monoclinic space group C2 with cell dimensions a = 100 Å, b = 60 Å, c = 62 Å, β = 116°. They contain one hexamer with R_6 _conformation in the asymmetric unit and have a solvent content of 50%. The crystal packing is shown in Figure 1c. Both the C2urea and C2scn structures have two zinc atoms/hexamer located 14.7 Å and 15.2 Å apart respectively. The zinc coordination is identical to the C222_1 _crystals. In the C2urea structure, two additional resorcinol molecules could be fitted into the electron density. The location of the first is very close to binding site II, described in [13]. At this site, the first hydroxy group of the resorcinol molecule is hydrogen bonded to the OG atom of SerB9 (2.9 Å) in an alternating conformation. The other hydroxyl group interacts with the carbonyl oxygen of GluB13 (2.5 Å) and a water molecule (3.1 Å). The water molecule, in turn, makes a hydrogen bond to the carbonyl oxygen of SerB9 (3.1 Å). In contrast to the phenolic binding interactions observed earlier in the PDB entry 1ZEG[13], where the phenolic oxygen hydrogen binds to HisB5, the angular orientation of the HisB5 in the C2urea structure does not seem to permit any interaction with the resorcinol molecule. The second additional resorcinol is located at the surface of the insulin in a solvent channel between two monomers, surrounded by water molecules. At the end of refinement, one glycerol molecule from the cryo-solution was added to both C2 structures. The glycerol molecule in the C2scn structure was found at a corresponding position as in the C222_1_scn structure, while the glycerol molecule in the C2urea structure was found in the solvent channel leading towards one of the zinc atoms, where it interacted with surrounding water molecules.

Refinement statistics for the four crystal forms is shown in Table 1. 95.4% of the residues in the C222_1_urea structure were found in the most favored regions of the Ramachandran plot and 4.6% in additional allowed regions. For the C222_1_scn structure, the corresponding values were 95.8% and 4.2% and for the C2 structures (C2urea/C2scn) 96.4%/96.0% and 3.6%/4.0%, respectively. The models showed no residues located in the generously allowed or disallowed regions of the plot.

**Table 1:** Data processing and refinement statistics for the orthorhombic and monoclinic insulin crystallized in presence of urea and thiocyanate respectively.

	Data sets			
	Urea containing crystals		Na-SCN containing crystals	
	C222_1_urea	C2urea	C222_1_scn	C2scn
*Data processing*				
Values in parenthesis are for the highest resolution shell				
Wavelength (Å)	1.0	1.1	1.3	1.3
Cell axis a, b, c (Å)	58.9, 219.3, 223.7	100.2, 60.2, 62.9	59.0, 219.5, 224.5	100.6, 60.8, 62.1
Cell angles α β γ (deg)	90.0 90.0 90.0	90.0 116.2 90.0	90.0 90.0 90.0	90.0 116.1 90.0
Temperature (K)	100	100	100	100
Diffraction limit (Å)	2.05	1.70	1.97	1.70
Highest resolution shell (Å)	2.1 – 2.05	1.75 – 1.70	2.0 – 1.97	1.75 – 1.704
No. of observations	450 276 (23593)	72 333 (3616)	885 954 (9682)	143 773 (4037)
Unique reflections	91 251 (6331)	34 195 (1952)	102 732 (3997)	34 924 (1890)
Redundancy	4.9 (3.7)	2.1 (1.8)	8.6 (2.4)	4.1 (2.1)
R_merge_^*a *^(%)	8.6 (39.3)	3.4 (16.9)	9.0 (37.3)	4.3 (13.3)
I/σ (I)	11.6 (3.2)	13.7 (4.1)	17.7 (2.4)	21.6 (6.5)
				
*Refinement statistics*				
Values in parenthesis are for the highest resolution shell				
Resolution range (Å)	28.31 – 2.05	19.57 – 1.70	19.99 – 1.97	19.78 – 1.70
Highest resolution shell (Å)	2.10 – 2.05	1.74 – 1.70	2.02 – 1.97	1.75 – 1.704
No. of reflections	86 749 (6376)	32 471 (1621)	97 508 (6629)	33 178 (1700)
Completeness	99.9 (100)	92.3 (62.5)	99.5 (93.1)	94.8 (67.5)
R value (%) ^*b*^	18.4 (21.3)	18.5 (22.9)	17.5 (21.7)	17.8 (25.5)
R_free _value (%) ^*b*^	22.7 (28.8)	22.3 (33.4)	21.1 (25.4)	22.1 (33.1)
				
r.m.s.d.^*c*^				
Bond length (Å)	0.016	0.012	0.014	0.011
Bond angles (deg)	1.6	1.3	1.4	1.3
				
B factors (Å ^2^) ^*d*^				
Average all atoms	32.2	27.2	33.8	20.9
All PheB1	40.1 (18)	27.4 (6)	42.4 (18)	18.7 (6)
α-helical PheB1	42.0 (7)	29.5 (3)	36.8 (5)	21.7 (3)
Non-helical PheB1	38.9 (11)	25.4 (3)	43.6 (13)	15.8 (3)

^*a *^R_merge _= S|I_i _- I|/SI where I_i _is an individual intensity measurement and I is the mean intensity for this reflection.

^*b *^R value = crystallographic R-factor = S|F_obs_| - |F_calc_|/S|F_obs_|, where Fobs and Fcalc are the observed and calculated structure factors respectively. R_free _value is the same as R value but calculated on 5% of the data not included in the refinement.

^*c *^Root-mean-square deviations of the parameters from their ideal values.

^*d *^Figures in parenthesis indicate number of monomers in each group.

All four insulin molecules are structurally very similar. Pair-wise superposition and comparison of the C2 structures results in a root-mean-square (r.m.s.) distance between corresponding C_α _atoms of 0.35 Å and of 0.88 Å when all common atoms are included. For the C222_1 _structures the same r.m.s. distances are 0.26 Å and 0.58 Å.

**Figure 2:**
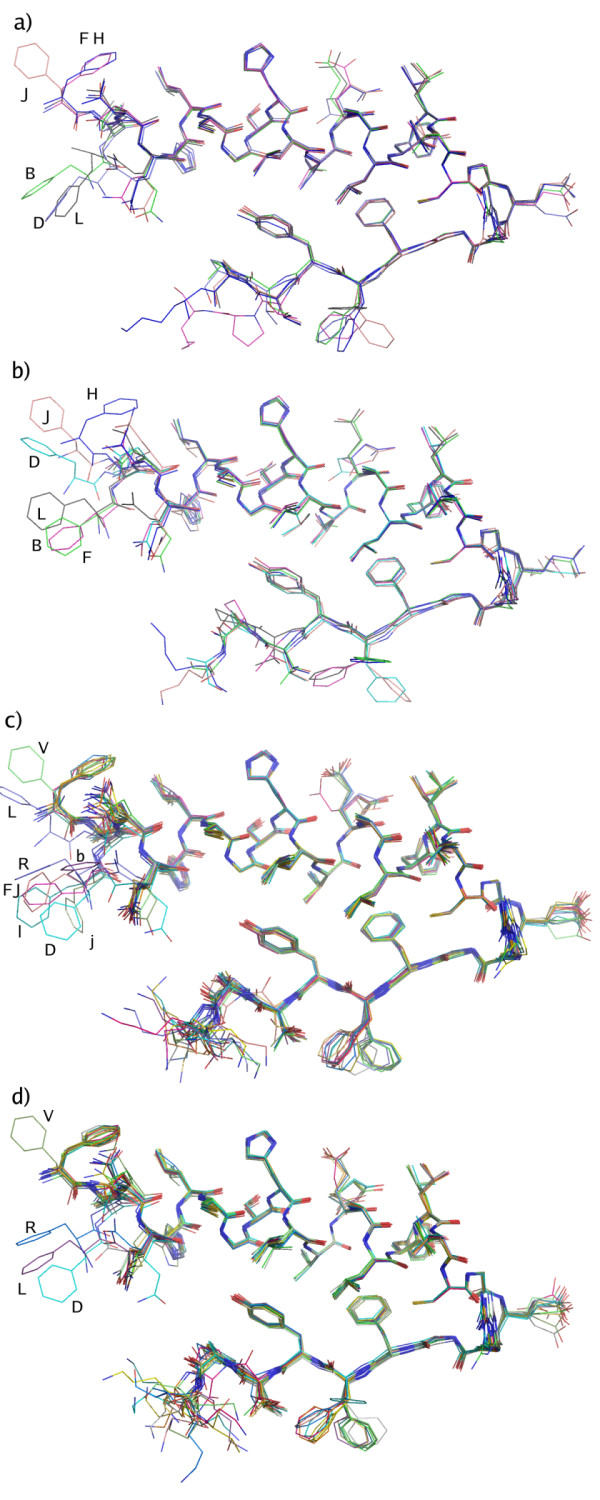
**Superposed B chains from the four structures**. (a) C2urea, (b) C2scn, (c) C222_1_urea and (d) C222_1_scn. Three B chains in each of the two C2 structures and the majority of the B chains in the C222_1 _structures have the PheB1 residue in an extended conformation (the top most population). Labels indicate chain names used in the final PDB files. For illustrative purpose, the side chain of the C-terminal LysB29 is included in the figures to illustrate the flexibility. This side-chain was subsequently omitted from several chains in the final PDB files due to disordered electron density.

A common feature of the four structures is the disruption of the otherwise characteristic continuous a-helix from reside B1 to B19. Instead of having a-helical conformation, some of the PheB1 residues in all four structures have a non-helical conformation. In the C222_1 _structures, the majority of the B-chains (11/18 and 14/18 in the C222_1_urea and C222_1_scn structures respectively) have this conformation (conformation I) where the phi/psi values of ValB2 are -80/+45. In the second conformation (II), the phi/psi values are ~-60/-45, closer to the typical values for an a-helix. The different B-chain conformations are illustrated in Figure 2, where they are superposed on each other. The distance between the Ca-atom of the PheB1 residue in the two different conformations is ~6 Å. For some of the residues, electron density could be seen for backbone atoms in more than one orientation. In such cases, the conformation with highest density was chosen, where also side chain atoms could be modeled with confidence. It should be noted that the density is weak for the side chain atoms of the Phe1 residue in chains B, F, b, f, h, j and l in the C222_1_scn structure and chains h, j, l in the C222_1_urea structure (chain names refer to the continuous naming convention of all chains in the PDB file). The PheB1 orientations in the C2 structures resemble those of the C222_1 _structures. Three out of the six B-chains in each structure have a non-helical conformation. The electron density is generally better defined in these two structures, which is reflected in the crystallographic B-factors. A comparison of the B-factors shows that PheB1 residues with non-helical conformation have a lower B-factor in three of the four structures, Table 1.

In contrast to the T_3_R_3_^f ^conformation, where B1–B3 have an extended conformation, only resides B1–B2 have a non-helical state. A similar, non-helical conformation of the PheB1 residue has previously been observed for one of the B-chains in an R_6 _insulin in complex with resorcinol (PDB ID: 1EVR) [11]. In that case, the carbonyl oxygen of PheB1 is coordinating a sodium ion which was further coordinated by the C terminal AsnA21 of a symmetry-related molecule. In our case, PheB1 is stabilized in a non-helical conformation by a hydrogen bond between amide nitrogen of PheB1 to the carbonyl oxygen of ThrA8 in a neighboring molecule, or a hydrogen bond between the carbonyl oxygen of PheB1 and the amide nitrogen of AsnB3 in the same chain. There are further interactions with symmetry-related molecules, such as the PheB1 amide nitrogen interactions with the OH group of a symmetry-related TyrA14, or the carbonyl oxygen of CysA20 and AsnA18.

In close proximity to the PheB1 residue of the three B chains with non-helical conformation in the C2urea structure there was an electron density peak with a height of 5 σ in the 2F_o_-F_c _map and ~5 σ in a F_o_-F_c _difference map. The location of the peak was close to the position where the carbonyl oxygen of PheB1 would be located if the conformation was a-helical. Given the observed electron density, a chloride ion was fitted into this position. It is coordinated to the amide nitrogen of HisB5 (3.2 Å) and two or three water molecules at an average distance of 3.3 Å. The corresponding sites in the C222_1_urea structure were too disordered to be interpreted in a similar manner.

Location of main-chain and side-chain atoms was ambiguous for the residues LysB29 and ThrB30 in most of the chains in the four structures. Furthermore, the following residues were modeled with alternating side chain conformations; C2urea structure: GlnB4.2, SerB9.5, ValB18.5, LeuB17.6; C2scn structure: GluB13.3, ValB18.4; C222_1_urea structure: LeuB17.I.1, ArgB22.I.4, GlnB4.I.6, ValB18.II.4 AsnB3.III.1; C222_1_scn structure: ValB18.I.2, ValB18.II.4, ValB18.II.5 (the roman numerals refer to the hexamer number while the single integer following a punctuation indicates monomer).

### Crystal packing

**Figure 3:**
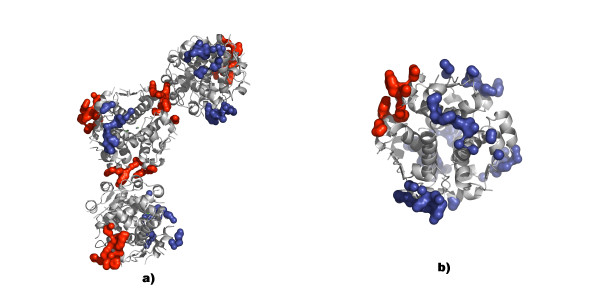
**Overview of the crystal contacts**. The crystal contacts in the C222_1 _(a) and C2 (b) crystals are shown in sphere representation where the interfaces comprising the tyrosine-tyrosine and glutamate-glutamate interaction are shown in red.

There is a strikingly high similarity between the crystallographic contact surfaces of the C222_1 _and C2 crystal forms. For five of the six contact sites found in the C2 crystal form, there is a corresponding contact surface with equivalent residue composition in the C222_1 _structure. Each hexamer in the C222_1 _structure has one symmetry-related contact surface that is identical to the hexamer-hexamer contact in the asymmetric unit. Including the hexamer-hexamer contacts within the asymmetric unit results in five such contact interfaces. In comparison, the C2 structure has in total six neighboring symmetry-related hexamers of which only one has the same kind of pair-wise interactions as the asymmetric hexamer-hexamer contact in the C222_1 _structure. An overview of the crystal contacts in the C222_1 _and C2 crystals is shown in Figure 3.

### A special crystal interaction at the dimer-dimer interface

**Figure 4:**
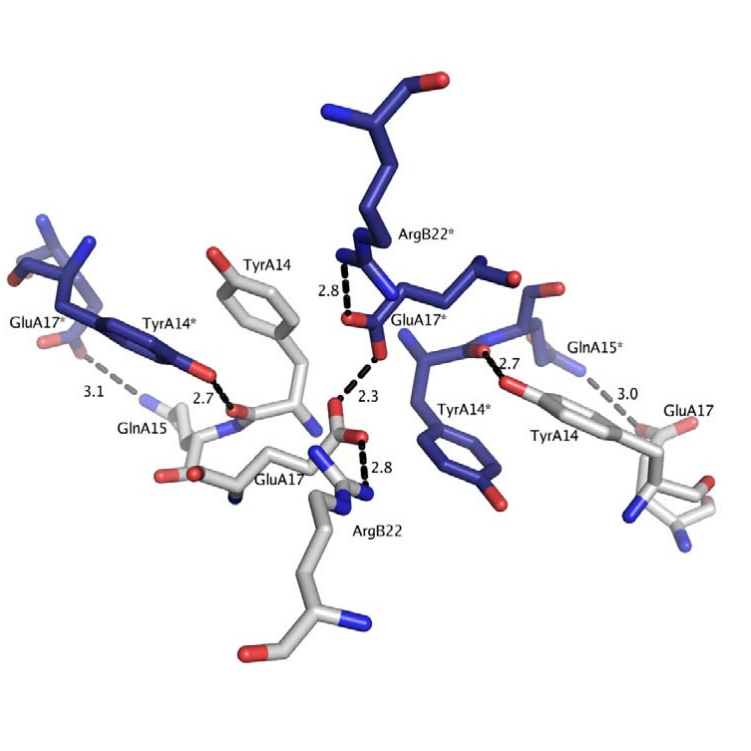
**One of the hexamer-hexamer interfaces in the C222_1 _urea structure**. The two tyrosine-tyrosine interactions (TyrA14-TyrA14) are flanking a close glutamate-glutamate contact of 2.3 Å. Both glutamates interact with ArgB22 (distance 2.8 Å). There are further two contacts between a GluA17 and GlnA15 from the neighboring hexamer. Residues from the neighboring hexamer are colored blue and marked with an *. Distances are given in Ångström (Å).

Each of the two hexamer-hexamer interfaces in the asymmetric unit of the C222_1 _crystals involves tyrosine-tyrosine interactions between different Tyr A14 groups. Tyr A14 is located at the dimer-dimer interface within the insulin hexamer so that the crystal packing brings four different Tyr A14 groups in proximity, Figure 4. The tyrosine side chains are pair wise stacked, such that the OH-group of TyrA14 in the first hexamer hydrogen bonds to the backbone oxygen of a TyrA14 in the neighboring hexamer (2.8 Å). The OH-group of the latter TyrA14 forms, in turn, hydrogen bonds to two water molecules. The polar interactions between the hexamers, Figure 4, comprise hydrogen bonds between GlnA15.I NE2 – GluA17.II OE2 (3.0 Å), GluA17.I OE1 – GlnA15.II NE2 (3.1 Å) (.I or .II denotes different hexamers). Additionally, there is an unusually short contact between two glutamates, GluA17.I OE1 – GluA17.II OE1 (2.32 ± 0.07 Å). In spite of the relative high pH of 6.5, the short Glu-Glu distance suggests a protonated carboxyl group of one of the glutamates. Normally, the pKa value for an exposed glutamate residue is ~4.4 in water environment. Given that GluA17 is protonated, the pKa value must thus be higher. One arginine (ArgB22) is located 2.8 Å from each glutamate and could potentially shift the pKa value by its inductive effect. The pK_a _value could also be shifted by the surrounding hydrophobic environment. GluA17 is flanked by the two tyrosine-tyrosine interactions, and it is conceivable that an uncharged protonated glutamate is more favorable in that environment. The short distance is indicative of a strong, low barrier hydrogen bond, where the proton is shared between the two carboxylates. Such low barrier hydrogen bonds have been found in protein active sites as part of enzyme catalysis [22,23] but also on protein surfaces [24].

As the pH is increased to 7.0, the second crystal form C2 appears. In this crystal form, there is only one crystal packing interaction comprising the tight glutamate-glutamate and tyrosine-tyrosine interaction, Figure 3. The increased pH could be the reason for the smaller number of such contacts. At higher pH, the shared hydrogen between the two glutamates becomes more delocalized and the repulsive forces will dominate. Consequently, the distance between the carboxylates is longer, 2.40 Å, versus 2.32 Å for the C222_1 _structures, indicating a weaker interaction at this pH. At pH values above 7.5, only the monoclinic P2_1 _crystal form [11] is observed, in which no such interface exists.

**Figure 5:**
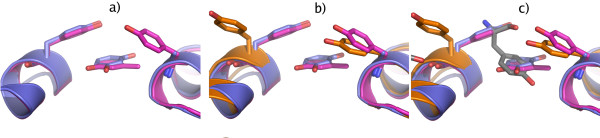
**Comparison of the binding pocket for a phenol derivative as seen in other structures and the position for hexamer-hexamer interaction as observed in this study**. In (a), the phenolic binding pockets of 1EVR (blue) and 1EV6 (purple) are superposed. One resorcinol and one meta-cresol molecule is shown to bind in the pocket created by the two flanking tyrosine residues. The side chain of the tyrosine to the right in 1EVR is missing in the pdb file. In (b) the same structures are superposed with the C222_1_urea structure (orange). The side chain of the left tyrosine is flipped to accommodate the hexamer-hexamer interaction shown in (c), where a neighboring hexamer from the asymmetric unit is included (grey). The tyrosine side chain of the second hexamer occupies the same position as the phenolic compounds.

Interestingly, the position occupied by the tyrosine from a neighboring hexamer is known to bind phenolic compounds like resorcinol and m-cresol [11,13]. In Figure 5a–c the phenolic binding sites in the pdb files 1EVR (R_6 _hexameric insulin complexed with resorcinol) and 1EV6 (R_6 _hexameric insulin complexed with m-cresol) [11] are compared with one of the hexamer-hexamer interfaces in the C222_1_urea structure. The phenyl ring of the neighboring tyrosine superposes the phenolic derivatives and should contribute to the stability of both the hexamer contact and the insulin structure.

### Analysis of crystal contact surfaces

In order to compare the different crystal forms of insulin, the contact sites were characterized by means of polarity and contact area. A summary of the properties for the various contact sites for the four structures presented in this study is shown in Table 2. Data for other crystal forms of hexameric insulin are also included. The surface area buried by crystal contacts range from 1423 Å ^2 ^to 3314 Å ^2^, which constitutes a fraction of buried surface area of between 10.6% and 24%. The smallest value is found for the orthorhombic C222_1 _crystals where the total contacts surface for the three hexamers is 4269 Å ^2^, which amounts to a contact surface of 1423 Å ^2^/hexamer. The largest surface area originates from the rhombohedral crystal form, space group R3 with T_6 _configuration of the B-chain, PDB ID: 1MSO [9]. The monoclinic crystals in space group C2 and P2_1 _as well as the tetragonal crystal in space group P4_3_2_1_2 all have six contact sites while the rest have eight. The size of individual contact sites ranges from 236 Å ^2 ^to 414 Å ^2^.

**Table 2:** Properties of crystal packing contact surfaces of insulin hexamers in seven different space groups.

**Protein**	**C2urea**	**C2scn**	**C2221urea**	**C2221scn**	**1EVR [11]**	**1EV3 [11]**	**1MSO [9]**	**1TRZ [17]**	**nph-ins**^*c*^
**Space group**	**C2**	**C2**	**C222**_1_	**C222**_1_	**P2**_1_	**R3**	**R3**	**R3**	**P4**_3_**2**_1_**2**
**B-chain configuration**	R_6_	R_6_	R_6_	R_6_	R_6_	R_6_	T_6_	T_3_R_3_^f^	R_6_
**pH**	7.0	7.0	6.5	6.5	6.7	8.5	6.3	6.4	7.3
**Solvent content. (%)**	50	50	64	64	49	40	36		48
**Avg B-factor**									
**protein atoms (Å**^2^**)**	25.5	19.5	32.1	33.5	41.1	30.2	12.0	32.8	34.8
**exposed atoms (Å**^2^**)**	27.9	21.5	34.0	35.9	42.5	34	12.3	34.7	36.5
**contact atoms (Å**^2^**)**	25.0	17.6	26.4	29.1	41.9	35.8	14.1	37.2	34.3
									
**Tot SASA (Å**^2^)^*a*^	14546	14478	40059	41532	15245	13573	13689	13904	15067
**A**_cont_**(Å**^2^)^*b*^	2117	2226	4269	4060	2113	2006	3313	2438	2279
**fract of tot SASA (%)**	14.6	15.4	10.6	9.8	13.9	14.8	24.2	17.5	15.1
									
**Atom type specific area as a fraction of A**_cont_									
**C area (%)**	55	56	53	53	41	45	46	50	48
**O area (%)**	25	24	31	32	33	35	37	32	38
**N area (%)**	21	20	16	15	27	20	18	16	14
**S area (%)**	0	0	0	0	0	0	0	2	0
									
**C aromatic (%)**	31	36	35	35	25	32	14	29	30
**C aliphatic (%)**	23	20	18	18	16	13	32	21	18
									
**Pos charged Area (%)**	5	3	3	3	5	0	16	16	2
**Neg charged Area (%)**	7	6	13	13	4	5	9	4	9

^*a*^*SASA = Solvent accessible surface area*.

^*b *^A_cont _= contact area between reference molecule and a symmetry related molecule.

^*c *^In house structure of human insulin co-crystallized with protamine.

The contact surfaces were characterized as either polar (oxygen and nitrogen atoms, including ionisable groups) or non-polar (carbons). The four structures presented in this study constitute a group with a high fraction of non-polar contact surface, ranging from 53% to 56% of the total contact area, compared to 41% to 50% for the other crystal forms. The monoclinic P2_1 _crystal form is the most hydrophilic, with a 40/60 distribution between hydrophobic and hydrophilic contact area. This analysis is limited in that bound water molecules were not considered in the crystal contact interactions since the criteria for modeling water molecules may vary among crystallographers and are also dependent on data quality. Several interactions could however involve hydrogen bonds to water molecules. Side chains with missing atoms were rebuilt in order to use the surface with an atom composition representing the true surface for the property calculations. They were however rebuilt automatically and could potentially be in a wrong orientation.

Comparing the residue identity of the crystal contacts for the crystals presented in Table 2 shows that seven of the interface residues are common for all crystal forms (GlnA5, ThrA8, TyrA14, GlnA15, AsnA18, TyrA19 and PheB1). Altogether, the contact sites for the six crystal types compared in this study cover almost the entire surface of an insulin hexamer. A comparison of the exposed residues with the residues involved in crystal contacts shows that all residues with an exposure of more than 20% participate in some contact interface. The degree of exposure was calculated according to [25]. A number of studies, where crystal packing contacts have been systematically investigated [26,27] conclude that atomic composition within crystal contacts is indistinguishable from that of the protein surface and is rather non-specific. Studies of pancreatic ribonuclease [28] and cutinase [29], crystallized in a number of space groups, showed in accordance with the present study that virtually the entirely protein surface can be involved in crystal contacts.

### Urea binding

**Figure 6:**
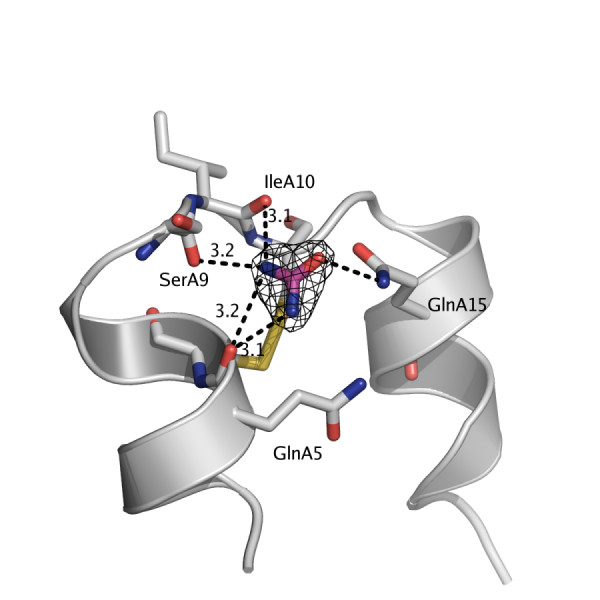
**Urea binding site**. The most commonly occupied binding site for the urea molecule in the C2urea and C222_1_urea structures. Hydrogen bonds are primarily directed towards the carbonyl oxygen GlnA5 but surrounding carbonyl oxygens from SerA9 and IleA10 are within reasonable distances. Marked distances are given in Ångström (Å).

The C222_1_urea and C2urea crystals were grown in presence of 3 M and 4 M urea, respectively. Seven urea molecules were built into the C2 structure. Five of these were located at equivalent positions in the monomers, Figure 6. The nitrogen atoms hydrogen bond primarily to the carbonyl oxygen of GlnA5, but the carbonyl oxygen of SerA9 and IleA10 are also within a reasonable hydrogen bonding distance (average 3.1 Å). In monomer six, the urea is either disordered or not present. Instead, a water molecule was built into the density. Nine out of 18 possible equivalent positions in the C222_1 _structure were occupied by urea. In the nine positions without a urea molecule, water was built in. Furthermore, these positions are more distant to a neighboring hexamer and therefore have a less well-defined electron density which may explain the inability to model a urea molecule. Exceptions from the above generalization are the monomers II.4 and III.1, which are close to a neighboring hexamer, but the electron density indicates two ordered water molecules.

Insulin has been shown to be tolerant of high concentrations of urea and other denaturants [30,31] and urea has previously been used to increase its solubility. One example is the inclusion of urea to promote growth of larger crystals of an insulin-protamine complex [32]. No significant conformational changes were detected in our structures as a result of direct urea interactions. In spite of its common use as a chemical denaturant of proteins, the molecular mechanism of urea-mediated unfolding is not known. Identification of denaturant interactions with proteins may give insight into the early stages of protein unfolding [33]. For a denaturant to be effective, protein-solvent interactions must be disturbed and this is thought to happen either through a direct or an indirect mechanism [34]. A direct mechanism would involve binding of urea molecules to the protein surface and thus compete with water-protein interactions and enhance the solubility of hydrophobic residues. Indirect urea denaturation would involve disruption of solvent-mediated hydrophobic interactions which would destabilize the protein structure. In addition, studies have shown that urea and guanidine hydrochloride at sub-denaturant concentrations stabilizes proteins at a sub global level in a mechanism called protein stiffening [35,36]. The present study shows that urea at concentrations ~3 M has one specific binding site on the surface of the insulin molecule, interacting with backbone carbonyl groups of primarily GlnA5 but also of SerA9 and IleA10 residues. Given the high concentrations of urea present in the crystallization experiments, we would expect to detect even weak binding sites with a K_d _of several hundred mM. Thus it seems unlikely that insulin denaturation occurs via a direct mechanism which requires binding of several urea molecules. On the other hand we see no signs of partial unfolding in our structures which would be indicative of an indirect mechanism. A recent study suggests that the denaturant effect of urea is neither due to a direct or indirect mechanism but rather an effect of a reduction of ion pairing between ionic and polar groups at aggregate surfaces [37], something which also could explain the relative higher fraction of non-polar surface at crystal contacts in our crystal forms.

## Conclusion

In spite of the extensive research on insulin crystallization during the last 80 years we could surprisingly identify two new crystal forms of human insulin. The major factor behind the change of space group from C222_1 _to C2 was an increase in pH from ~6.5 to 7.0. A comparison of the crystal contacts in the two space groups showed that the interfaces are very similar. The most obvious difference and the most remarkable contact interaction was the double TyrA14-TyrA14 interaction combined with a tight GluA17-GluA17 interaction. When taking all symmetry-related contacts into account it was found that this contact type was more frequent in the C222_1 _crystals when compared to the C2 crystals. This crystal packing interaction has not been observed in any of the previously known insulin crystals. The addition of chaotropes such as urea and thiocyanate to the crystallization buffer can have an effect on the protein charge properties by charge screening. This could explain altered pK_a _values of the glutamic acids involved in the crystal contacts and the higher fraction of hydrophobic crystal contacts in the present crystal forms compared to previously known insulin crystals. The short carboxyl-carboxyl interaction indicates the presence of a shared proton between the two groups and would be a strong low barrier hydrogen bond which should contribute to the stability of the crystals.

In the case of insulin, much effort has been put into the modification of the dimerization interactions [38,39] and hexamer formations [40,41], but less focus has been on the inter-hexamer interactions in solution or within crystals. Modifications of surface residues can induce changes in crystal packing due to breaking of existing interactions and/or formation of new ones [42,43]. Engineering of the protein surface to specifically induce a change in the crystal form or improve stability in lattice contacts may produce a better diffracting crystal [44]. Since insulin is a therapeutic protein administered also in crystalline form, the discovery and analysis of new polymorphic forms has implications beyond providing improved crystals for structural studies. The inter-hexamer interaction found in the present structures provides an interesting and novel interface that is specific for these two crystal forms. A single additional hydrophobic or several polar interactions may increase the half-life of a protein by several orders of magnitude [45]. Thus, additional inter-hexamer interactions can increase the stability and thus the shelf life of crystalline insulin formulations. The structures presented here provide a framework for further site-directed mutagenesis studies of the residues involved in inter-hexamer interactions, aimed at providing improved formulations useable within the rapidly advancing field of alternate delivery routes of crystalline biopharmaceuticals [3].

This study also demonstrates the usefulness of X-ray powder diffraction (XRPD) on protein samples. The small size of the initially obtained micro crystals made visual analysis and single crystal X-ray diffraction difficult. However, the combination of XRPD and principal component analysis (PCA) facilitated the identification of a new crystal form [21]. Since the use of proteins as therapeutic agents is a growing field, applications of protein XRPD, similar to the present study, will have an important role during discovery and development of therapeutic protein formulations.

## Methods

### Crystallization

Human insulin was obtained from Novo Nordisk A/S (Denmark). Crystals were grown by hanging-drop vapor diffusion technique at 291 K. The crystals were obtained in crystallization experiments with varying reservoir concentrations of NaCl and urea. A protein solution containing 6.9 mg/ml of human insulin, with zinc content corresponding to two zinc ions per hexamer and 50 mM resorcinol were mixed with equal volumes of reservoir solution. The protein solution was filtered through a 0.22-micrometer centrifugal filter (Ultrafree-MC, Millipore, USA) prior to crystallization. The best diffracting crystals were obtained from the following conditions. For the C222_1 _crystals: 2 M NaCl, 3 M urea, 100 mM phosphate buffer pH 6.5; for the C2 crystals: 2.5 M NaCl, 4 M urea, 100 mM phosphate buffer pH 7.0. The same two crystal forms were obtained when including 15 mM NaSCN in the protein solution in absence of urea and using a reservoir solution containing 5% (v/v) ethanol and 200 mM phosphate buffer at pH 6.5 and 7.0, respectively. Crystals with dimensions of about 0.25 mm on one edge were detected after two days. The two orthorhombic crystals in space group C222_1 _diffracted to a resolution of 2.0 Å while data from the crystals in space group C2 could be collected to 1.7 Å.

### Data collection and refinement

Data sets were collected from a single crystal of each type at 100 K using synchrotron radiation (Maxlab synchrotron, Lund, Sweden, beamline 911-2 and 911-3 [46]), with a MarMosaic 225 CCD detector (MarResearch, Evanston, USA). The urea containing crystals were soaked in a cryo-solution containing 23% glycerol and 77% reservoir prior to freezing in liquid nitrogen. For the NaSCN crystals a cryo-solution containing 30% glycerol was needed. All data sets were processed and scaled using the XDS package [47].

For the C222_1 _crystals with urea, an additional low-resolution data set was collected from the same crystal and merged with the high-resolution data. An in house structure of a hexamer with R_6 _conformation, excluding all non-protein atoms except zinc, was used as search model for molecular replacement in Molrep [48]. Three hexamers were found in the asymmetric unit, corresponding to a solvent content of 64%. During refinement in Refmac [49], 5% of the data was excluded and used for calculation of the R-free value. The initial 2F_o_-F_c _map clearly indicated positions of chloride ions and resorcinol molecules (six in each hexamer). After several rounds of refinement using the maximum likelihood option in Refmac and manual adjustments of main-chain and side-chain atoms in Coot [50], TLS refinement [51] was employed, with each monomer defined as a separate TLS group. Subsequently, water was added by the find-water function in Coot. At the end of the refinement, urea molecules were modeled into the electron density using positive F_o_-F_c _peaks, where the shape of the 2F_o_-F_c _density was flat and reminiscent of the triangular shape of a urea molecule. 14 such positions were found with an average B-factor of 53.1 Å ^2^. The final number of water molecules was 634 with an average B-factor of 41.7 Å ^2^.

The C2 structure obtained with urea was solved with the same search model as the C222_1 _data set and the procedure for structure solving and refinement followed the same route. In the beginning of the refinement, there was a clear 2F_o_-F_c _density for an extra resorcinol binding site, and at later stages of refinement, a second additional position showed density resembling a resorcinol molecule. Seven urea molecules (average B-factor = 39.3 Å ^2^) and 257 water molecules were built into the density at the end of refinement.

For the two structures co-crystallized with NaSCN, one high and one low resolution data set were collected and subsequently merged. The structures were solved using the urea containing structures as search models in molecular replacement rounds (excluding non-protein atoms except zinc). Based on the experience of flexible residues in the B-chain terminals, the search models were truncated at both ends to reduce bias (PheB1, ValB2, LysB29 and ThrB30). Refinement followed the same scheme as for the urea crystals. In total, six resorcinol molecules were modeled into the C2 structure and 18 in the C222_1 _structure (six in each hexamer). 313 water molecules were fitted into the C2 structure and 755 into the C222_1_. Based on the shape of the electron density, each zinc atom was found to interact with one thiocyanate molecule. Data collection details and refinement statistics for all four structures are summarized in Table 1. In this paper, the crystals obtained in presence of urea will be referred to as C222_1_urea and C2urea whilst the two forms obtained with thiocyanate are referred to as C222_1_scn and C2scn.

### Analysis of crystal packing

Symmetry-related molecules were generated in Pymol [52] for analysis of crystal contacts. A rather strict criterion for identification of symmetry contacts was used by searching for symmetry-related atoms within 4 Å from the reference protein. In order to limit the analysis to protein-protein interactions, non-protein atoms such as water and urea molecules were removed prior to searches. Residues with missing side-chain atoms were reconstructed in Swiss-PdbViewer [53] using the 'auto reconstruct residues with missing atom' function. When dual conformations of residue side chains were present, the one with highest occupancy, or when equal, the conformation denoted with an 'A' in the pdb file was chosen. The contact area (A_cont_) between the reference molecule and neighboring symmetry-related molecules was defined as the solvent accessible area buried by symmetry-related molecules. The solvent accessible surface area (SASA) was calculated in areaImol (within the CCP4 program package [54]) that utilizes the algorithm of Lee & Richards [55]. A water probe of 1.4 Å was used. A_cont _for each atom was obtained by taking the SASA for the reference molecule alone, minus the SASA when contact atoms were present. All atoms with reduced SASA were assumed to be involved in contacts with a symmetry molecule. Basic physicochemical properties related to hydrophobic surface area were calculated for the atoms of the reference molecule involved in contacts. Contact areas were split into hydrophobic (carbons) and hydrophilic (nitrogen and oxygens), aliphatic-, aromatic carbons and positive-, negative charged area. For the sake of simplicity and ease of comparison, crystals without a full hexamer in the asymmetric unit were complemented with the required symmetry molecules by applying appropriate symmetry operations to generate a hexamer prior to calculations. A hexamer was considered as one molecule.

Coordinates with structure factors have been deposited to the Protein Data Bank (PDB) [56] with the accession codes 2OLY, 2OLZ, 2OM0 and 2OM1.

## Authors' contributions

MN collected and interpreted the data and drafted the manuscript. GS conceived the study and participated in the crystallization experiments and interpretation of results. Both authors read and approved the final version of the manuscript.
